# The genus *Diplocheila* Brullé, 1834 in Cambodia, with descriptions of two new species (Coleoptera, Carabidae, Licinini)

**DOI:** 10.3897/zookeys.1044.60072

**Published:** 2021-06-16

**Authors:** Gianni Allegro, Pier Mauro Giachino

**Affiliations:** 1 Strada Patro 11, I-14036 Moncalvo (AT), Italy Unaffiliated Moncalvo Italy; 2 Via della Trinità 13, I-10010 San Martino Canavese (TO), Italy Unaffiliated San Martino Canavese Italy

**Keywords:** *Diplocheila erwini* sp. nov., *Diplocheila walterrossii* sp. nov., faunistics, Oriental Region, taxonomy

## Abstract

The *Diplocheila* species recorded from Cambodia are discussed and two new species, *Diplocheila walterrossii***sp. nov.** and *D. erwini***sp. nov.** are described. Moreover, the holotypes of *D. laevigata* (Bates, 1892) and *D. laevigotoides* Jedlička, 1936, two often misinterpreted species from the Oriental Region, are illustrated and some aspects of their morphology are clarified. Finally, an analytical key to all species recorded from Cambodia is provided.

## Introduction

The carabid fauna of Cambodia is still poorly known. Only 172 species belonging to the family Carabidae are recorded from this Asian country to date (Choi et al. 2019; Anichtchenko et al. 2020), and very few systematic studies have been devoted to this fauna in the recent past. This is probably due to the long-standing political instability suffered by Cambodia until the 1990s, which to a large extent has hindered entomological investigations in the field.

In recent years Walter Rossi, a world-renowned specialist in entomoparasitic fungi, carried out various expeditions in Cambodia, collecting a large amount of entomological material (mostly by light trapping) which was distributed to world specialists for identification. We are grateful to him for the gift of the Carabidae specimens, which are currently under study. We have no doubt that these studies will lead to a large increase in the number of species recorded from this country, as well as the likely discovery of many new species.

This paper concerns the species belonging to the genus *Diplocheila* Brullé, 1834 collected in Cambodia by W. Rossi, including the descriptions of two new species: *Diplocheila walterrossii* sp. nov. and *Diplocheila erwini* sp. nov. In addition, the holotypes of *Diplocheila laevigata* (Bates, 1892) and *Diplocheila laevigotoides* Jedlička, 1936, two species often misinterpreted in the past, have been examined and illustrated. Finally, a key to all species of the genus currently recorded from Cambodia is provided.

## Materials and methods

The specimens studied or mentioned in the text are deposited in the following museums and private collections:

**BMNH**The Natural History Museum, London, United Kingdom;

**MCSNG** Museo Civico di Storia Naturale “Giacomo Doria”, Genova, Italy;

**NMPC**National Museum of Natural History, Prague, Czech Republic;

**CAl** Gianni Allegro Collection, Moncalvo (AT), Italy;

**CCa** Achille Casale Collection, Torino, Italy;

**CGi** Pier Mauro Giachino Collection, San Martino Canavese (TO), Italy.

The abbreviations used for the type material are as follows:

**HT** holotype.

**PT (PTT)** paratype (paratypes).

The type locality is quoted in the original label form.

**Figure 1.**
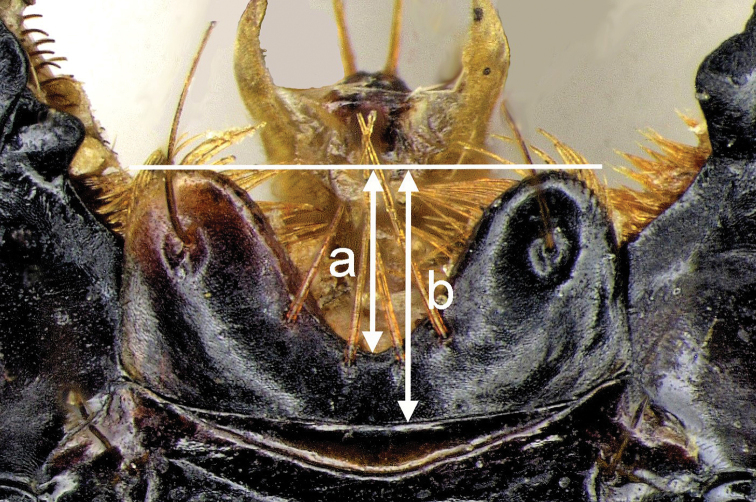
Sketch of the *Diplocheila* labrum-clypeal area showing the intervals measured to calculate labrum ratio (LR) = a/b.

Apparent body length (**ABL**) is measured from the apex of labrum to apex of the longer elytron. **PW**: pronotum width at the widest point; **PL**: pronotum length measured from the apical to basal margin along midline; **EW**: elytral width at the widest point; **EL**: elytra length from the base of scutellum to apex of the longer elytron; **LR**: ratio of the length measured between the straight line connecting the apices of lateral lobes of the labrum and narrowest point of medial emargination (a) and the length measured between the straight line connecting the apices of lateral lobes of the labrum and frontoclypeal suture (b) (Fig. 1).

Digital images were taken using a Leica DFC295 camera mounted on a Leica M205 C stereomicroscope and using Leica Application System v. 4.0 software.

## Taxonomy

The systematic arrangement of *Diplocheila* is still unclear. In particular, the attribution of the species to subgenera is still under discussion, as well as the validity of the subgenera (Will 1998). Ball (1959, 1966, 1992) first proposed a comprehensive classification of world species into subgenera (*Diplocheila* s. str., *Neorembus* Ball, 1959 and *Isorembus* Jeannel, 1949); moreover, he recognized two species groups within *Diplocheila* s. str. (*polita* and *daldorfi* groups) and three inside *Isorembus* (*aegyptiaca*, *striatopunctata*, and *zeelandica* groups). Lorenz (2005), in the list of extant ground beetles of the world, referred to the arrangement by Ball (1959), but with a different subdivision of species into subgenera; Lafer and Kataev (2008) accepted Ball’s approach in the subdivision of species but proposed to reinstate the subgenus Submera Habu, 1956, which had been treated by Ball (1959) as a synonym of *Isorembus*; in the same way, Huber and Marggi (2017), in the recent catalogue of Palaearctic Coleoptera, reinstated the subgenus Submera as valid, following a different subdivision of species compared to Ball’s arrangement. Unfortunately, the classifications proposed by the catalogues of Lorenz (2005) and Huber and Marggi (2017) lack authors’ comments explaining their treatment. In this context, we prefer to follow Ball’s arrangement as the most justified, but we accept the proposal by Lafer and Kataev (2008) to retain valid the subgenus Submera for *Diplocheila laevis* (Lesne, 1896).

According to CarabCat, the Global database of ground beetles (Lorenz 2020), four *Diplocheila* species are recorded so far from Cambodia: *Diplocheila latifrons* (Dejean, 1831), *Diplocheila distinguenda* (LaFerté-Sénectère, 1851), *Diplocheila laevigata* (Bates, 1892), and *Diplocheila colossus* (Bates, 1892), although literature references were not provided. We were able to find references to the presence in Cambodia of *D. latifrons*, *D. laevigata*, and *D. colossus* in Andrewes (1922), whilst *D. distinguenda* is listed in the species from Cambodia by Anichtchenko et al. (2020).

In the material collected by Walter Rossi in Cambodia we have identified:

Diplocheila (Neorembus) latifrons (Dejean, 1831)

Diplocheila (Submera) laevis (Lesne, 1896) (new record for Cambodia)

Diplocheila (Diplocheila) laevigata (Bates, 1892)

Diplocheila (Diplocheila) erwini sp. nov.

Diplocheila (Diplocheila) walterrossii sp. nov.

### Diplocheila (Neorembus) latifrons ssp. latifrons

Taxon classificationAnimaliaColeopteraCarabidae

(Dejean, 1831)

662CE56D-E2A7-59D8-894A-C18070578185

Figures 25 , 32

Rembus latifrons Dejean 1831: 679
Rembus opacus Chaudoir 1852: 67
Rembus opacus Chaudoir, 1852: Bates 1873: 265
Rhembus opacus (Chaudoir, 1852): Bates 1889: 267
Rhembus latifrons (Dejean, 1831): Bates 1892: 327
Diplochila latifrons (Dejean, 1831): Lesne 1904: 72
Diplochila latifrons (Dejean, 1831): Andrewes 1922: 283
Diplocheila latifrons (Dejean, 1831): Andrewes 1930: 150
Submera latifrons (Dejean, 1831): Habu 1956: 61
Diplocheila latifrons (Dejean, 1831): Ball 1959: 41
Diplocheila latifrons (Dejean, 1831): Kryzhanovskij et al. 1995: 159
Diplocheila latifrons (Dejean, 1831): Lorenz 2005a: 343
Diplocheila latifrons (Dejean, 1831): Lorenz 2005b: 580
Diplocheila latifrons (Dejean, 1831): Lafer and Kataev 2008: 684
Diplocheila latifrons (Dejean, 1831): Huber and Marggi 2017: 626

#### Type locality.

Oriental India.

#### Material examined.

Cambodia: 1 ♂ Kampong Chhnang Province, Khom Domnatpopol, Tonle Sap Lake, 21.V.2018, Rossi, Bernardi and Kong leg. (CAl); 1 ♀ Lamphun, Mueang Lamphun District, near Umong, 24.II.2017, W. Rossi and V. Kong leg. (CAl); 1 ♂ Banteay Meanchey, near Sisophon, campus of the Mean Chey University, 1.XI.2018, W. Rossi and V. Kong leg. (CAl);1 ♂ Banteay Meanchey, near Sisophon, campus of the Mean Chey University, 20.V.2019, W. Rossi and V. Kong leg. (CGi); 1 ♀ Mean Chey, 20.V.2019, W. Rossi and V. Kong leg. (CAl); 1 ♀ Kampong Chhnang, Rolea B’ier District, Ourung Village, 20–23.V.2018, Rossi, Bernardi and Kong leg. (CGi).

Thailand: 1 ♂ 1 ♀ Chiang Mai, 6.V.1988 (CGi). India: 1 ♀ Uttar Pradesh, Jhansi District, Babina, VIII.1987 (CGi).

#### Remarks.

*Diplocheila latifrons* is the only species belonging to subgenus Neorembus. Two subspecies are known: the nominotypical one, which is widely distributed across China, Korea, Japan, India, Myanmar, the Russian Far East, Vietnam, Laos, Cambodia, Thailand and Indonesia, and the ssp. darlingtoni Ball, 1959, which is only recorded from the Philippines (Andrewes 1922; Ball 1959).

Head, base of elytra, and aedeagus are also illustrated by Lafer and Kataev (2008).

### Diplocheila (Submera) laevis

Taxon classificationAnimaliaColeopteraCarabidae

(Lesne, 1896)

3C1B7F50-06C7-5DE7-90EA-ECA39CF1CC62

Figures 24 , 31

Rhembus laevis Lesne 1896: 243
Rhembus laevis Lesne, 1896: Bouchard 1903: 171
Diplochila laevis (Lesne, 1896): Lesne 1904: 72
Diplochila laevis (Lesne, 1896): Andrewes 1922: 284
Diplocheila laevis (Lesne, 1896): Andrewes 1930: 150
Diplocheila laevis (Lesne, 1896): Ball 1959: 52
Diplocheila laevis (Lesne, 1896): Lorenz 2005a: 343
Diplocheila laevis (Lesne, 1896): Lorenz 2005b: 571
Diplocheila laevis (Lesne, 1896): Lafer and Kataev 2008: 690
Diplocheila laevis (Lesne, 1896): Huber and Marggi 2017: 626

#### Type locality.

Bangkok (Le P. Larnaudie); Chantaboun à Battambang (Siam Cambodgien); Meuwen Bay (Java).

#### Material examined.

Cambodia: 1 ♂ Kampong Chhnang, banks of Tonle Sap Lake, 17.V.2019, W. Rossi and V. Kong leg. (CAl); 1 ♂ Kampong Chhnang Province, Sankat Kampong Chhnang, Phum Toul Ompel, banks of a branch of Tonle Sap Lake, 12°14'N, 104°41'E, 4.XI.2018, W. Rossi and V. Kong leg. (CGi).

#### Remarks.

*Diplocheila laevis* is widely distributed across South-East Asia, China, Indonesia, and the Philippines (Andrewes 1922; Ball 1959; Lafer and Kataev 2008). The aedeagus is also illustrated by Lafer and Kataev (2008). As far as we know, this is the first record of the species from Cambodia.

### Diplocheila (Diplocheila) laevigata

Taxon classificationAnimaliaColeopteraCarabidae

(Bates, 1892)

382C4F21-4C4D-5D4F-801E-642E5B814422

Figures 2 , 6 , 8 , 12 , 14 , 18 , 20 , 26 , 27

Rembus politus MacLeay 1825: 16
Rembus politus MacLeay, 1825: Redtenbacher 1867: 10
Rhembus laevigatus Bates 1892: 326
Eccoptogenius moestus Bates 1889: 267
Eccoptogenius moestus Bates, 1889: Andrewes 1921: 176
Diplochila laevigata (Bates, 1892): Andrewes 1921: 177
Diplochila laevigata (Bates, 1892): Andrewes 1922: 282
Diplocheila laevigata (Bates, 1892): Andrewes 1930: 149
Diplocheila laevigata (Bates, 1892): Habu 1956: 55
Diplocheila laevigata (Bates, 1892): Ball 1959: 35
Diplocheila laevigata (Bates, 1892): Lorenz 2005a: 342
Diplocheila laevigata (Bates, 1892): Lorenz 2005b: 571
Diplocheila laevigata (Bates, 1892): Lafer and Kataev 2008: 682
Diplocheila laevigata (Bates, 1892): Huber and Marggi 2017: 626

#### Type locality.

Kawkareet in Tenasserim (Myanmar).

#### Material examined.

**Figure 2.**
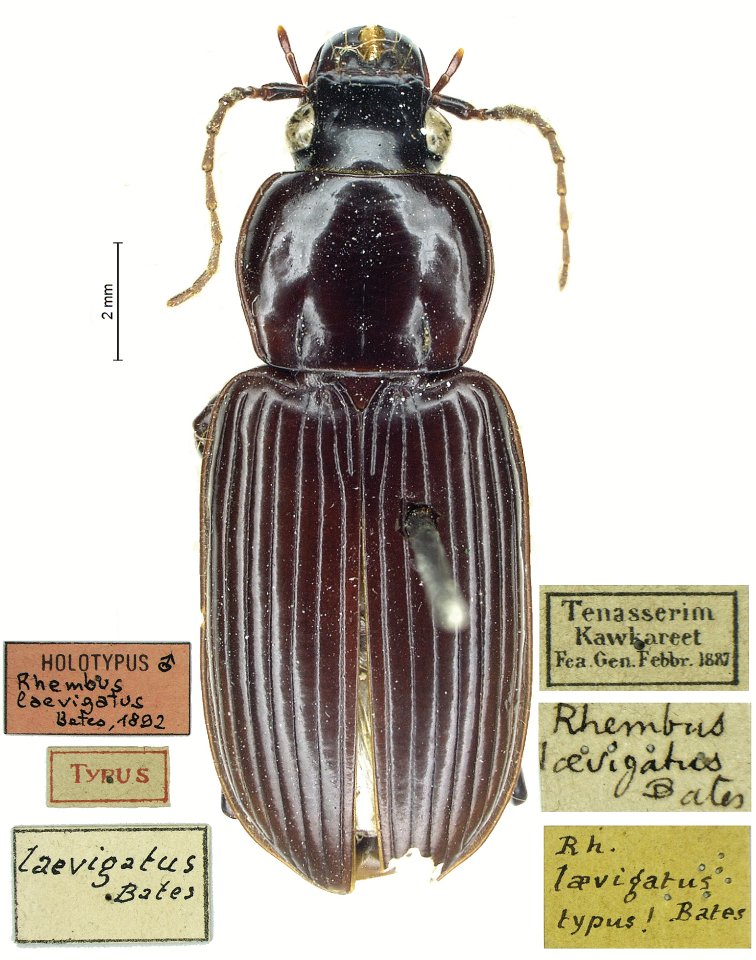
*Diplocheila laevigata* (Bates, 1892): habitus of holotype.

Myanmar: HT ♂, Kawkareet in Tenasserim, Gen. Febbr. 1887, Fea legit (MCSNG) (figs 2, 6, 12, 26). Cambodia: 3 ♂♂ 2 ♀♀ Kampong Chhnang, banks of Tonle Sap Lake, 17.V.2019, W. Rossi and V. Kong leg. (CAl, CGi); 1 ♂ Kampong Chhnang, Rolea B’ier District, Toekchenh Village, 18.V.2019, W. Rossi and V. Kong leg. (CAl);1 ♂, Khsam, Kampong Chhnang, 12°16'47"N, 104°39'28.6"E, 29.XI–3.XII.2019, light trap, W. Rossi and V. Kong leg. (CAl).

#### Diagnosis.

*Diplocheila laevigata* may be distinguished by the combination of the following characters. ABL = 14–16 mm; head with 1 supraorbital setiferous pore on each side; labrum with 6 setae (4 medial + 2 lateral), symmetrical and moderately emarginate (LR = 0.63–0.65) (Figs 12, 14); anterior margin of clypeus hardly concave (Fig. 12); pronotum transverse (PW/PL = 1.29–1.31), with sides delicately sinuate backwards (Fig. 6); elytral striae distinctly punctate; apical lamella of the median lobe of aedeagus in dorsal view shortly triangular, with blunt apex (Figs 20, 26).

#### Remarks.

*Diplocheila laevigata* is recorded from southern China, Myanmar, Vietnam, Laos, and Cambodia (Andrewes 1922; Ball 1959; Lafer and Kataev 2008). Unfortunately, the male HT in the Fea Collection at MCSNG is an immature specimen (Fig. 2) with a scarcely chitinized aedeagus (Fig. 26); however, its examination, made possible thanks to the courtesy of the Honorary Curator Roberto Poggi, revealed that it was conspecific with the specimens from Cambodia (Figs 8, 14, 27). An aedeagus of *D. laevigata*, substantially corresponding to the HT, is also illustrated by Lafer and Kataev (2008).

Two specimens from Thailand deposited in CGi (2 ♂♂, Chiang Mai, 6.V.1988, R. Sciaky det.; Figs 9, 15) show a similar aedeagal morphology but pronotum and labrum are differently shaped compared to the HT and to the specimens from Cambodia; they could belong to a new species, but it is not described here, awaiting more abundant material. A further specimen from Thailand deposited at BMNH (1 ♀, Bangkok, Larnaudie leg., H.E. Andrewes Coll.) probably belongs to a different, new species.

A male specimen from Indonesia deposited at BMNH (1 ♂, Indes Neerl., Boucard leg., H.E. Andrewes Coll.) differs from the *D. laevigata*HT not only in external morphology, but also in the shape of the aedeagus (although this is damaged, the apical lamella appears to be clearly different); its external morphology does not match *D. laevigotoides*HT either, in spite of the opinion of Lafer and Kataev (2008) that the records of *D. laevigata* from Indonesia, together with those from Japan and the Philippines, are probably instead *D. laevigotoides*. Therefore, this specimen also likely belongs to a new species, awaiting description when more abundant material becomes available.

### Diplocheila (Diplocheila) laevigotoides

Taxon classificationAnimaliaColeopteraCarabidae

Jedlička, 1936

900ED10A-F380-53AA-AD0A-9152CA9D3958

Figures 3 , 7 , 13

Diplochila laevigotoides Jedlička 1936: 92
Diplocheila laevigatoides Jedlička, 1936: Ball 1959: 36
Diplocheila laevigotoides Jedlička, 1936: Lorenz 2005a: 342
Diplocheila laevigotoides Jedlička, 1936: Lorenz 2005b: 571
Diplocheila laevigatoides Jedlička, 1936: Lafer and Kataev 2008: 682

#### Type locality.

The Philippines: Manila.

#### Material examined.

**Figure 3.**
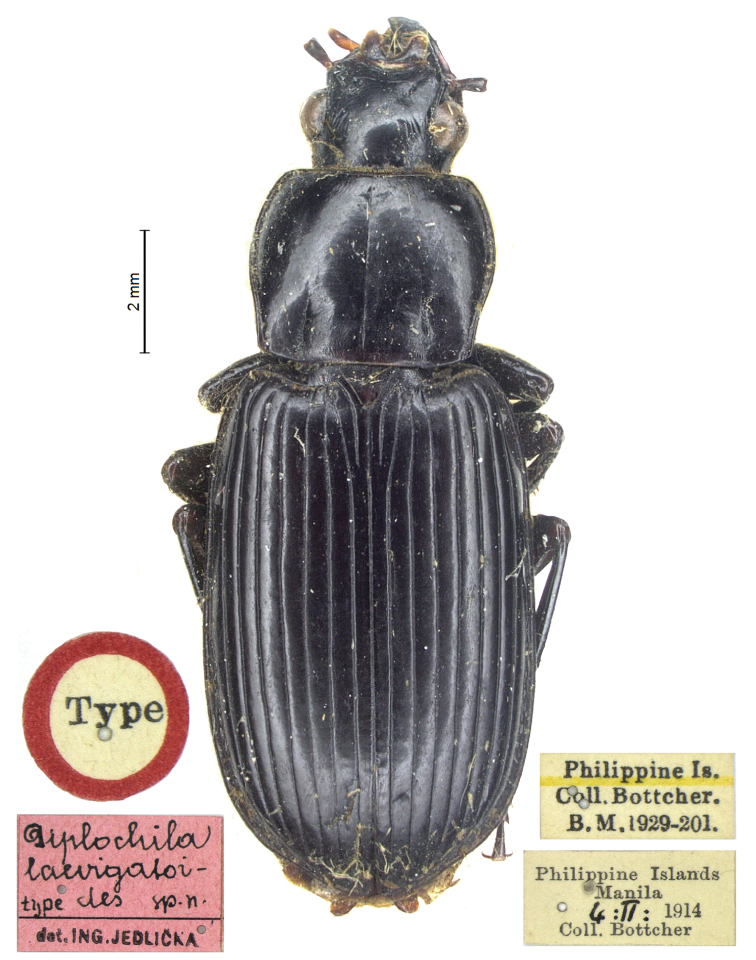
*Diplocheila laevigotoides* Jedlička, 1936: habitus of holotype.

Philippine Islands, ♀ HT, Manila, 4.II.1914, Coll. Bottcher (BMNH) (Fig. 3).

#### Diagnosis.

*Diplocheila laevigotoides* may be distinguished by the combination of the following characters. ABL = 14 mm; head with 1 supraorbital setiferous pore on each side; labrum with 6 setae (4 medial + 2 lateral), symmetrical and deeply emarginate (LR = 0.80) (Fig. 13); anterior margin of clypeus markedly concave (Fig. 13); pronotum transverse (PW/PL = 1.31), with sides markedly sinuate backwards (Fig. 7); elytral striae nearly impunctate.

#### Remarks.

*Diplocheila laevigotoides* has often been confused in the past with *D. laevigata*; for this reason, although the species is probably not present in Cambodia, we decided to examine and illustrate the habitus of the HT specimen (Fig. 3) deposited at BMNH, in order to examine some aspects of its morphology and clarify possible misunderstandings. For this reason, the species is also included in the identification key to the species of Cambodia (see below). Unfortunately, the HT is a female specimen (and the unique PT specimen found by Jiří Hájek in Jedlička Collection at NMPC is also a female). Nevertheless, its habitus and the particular shape of the pronotum and labrum allow for *D. laevigata* and *D. laevigotoides* to be reliably distinguished. The opinion of Lafer and Kataev (2008) that the records of *D. laevigata* from Indonesia could refer to *D. laevigotoides* is not confirmed by our examination of a male specimen from the same area (see Remarks under *D. laevigata*). We have nothing at present to add about the records from Japan (Habu 1959) and the Philippines (Ball 1959), which are also suspected to belong to *D. laevigotoides*.

It is curious that this species was described by Jedlička (1936) as *D. laevigotoides* (probably a printing error), although the label of the HT reports “*laevigatoides*” (Fig. 3).

### Diplocheila (Diplocheila) walterrossii sp. nov.

Taxon classificationAnimaliaColeopteraCarabidae

582C63BB-E91F-5B48-8E69-73704D0409DA

http://zoobank.org/714A6638-4FF9-4169-AC5F-E3F937247446

**Figure 4.**
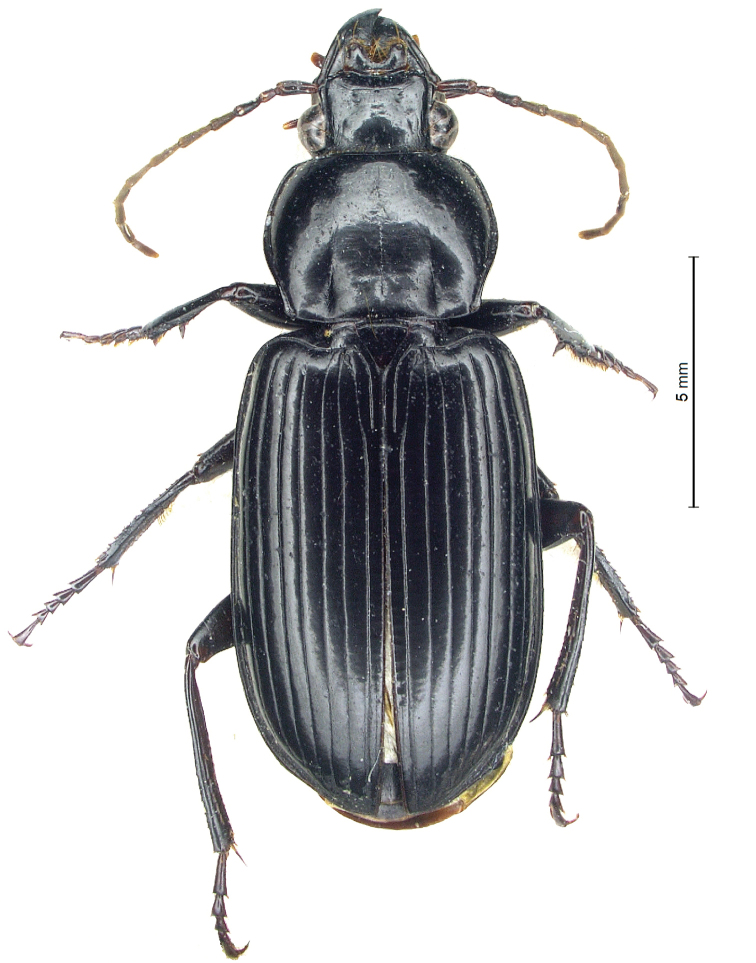
*Diplocheila walterrossii* sp. nov.: habitus of holotype.

Figures 4 , 10 , 16 , 19 , 22 , 29 , 33

#### Type locality.

Cambodia, Siem Reap Province, N Siem Reap City, 13°26'29"N, 103°52'25"E.

#### Material examined.

***Holotype***: ♂, Cambodia, Siem Reap Province, N Siem Reap City, 13°26'29"N, 103°52'25"E, light trap, 13.XI.2018, W. Rossi and V. Kong leg. (CGi).

***Paratypes***: (7 ♂♂ and 3 ♀♀); 2 ♂♂, same data as HT; 2 ♂♂ Cambodia, Kampong Chhnang Province, Khom Domnatpopol, Tonle Sap Lake, 12°14'14"N, 104°41'15"E, light trap, 21.V.2018, Rossi, Bernardi and Kong leg.; 1 ♂ 1 ♀, Cambodia, Kampong Chhnang, banks of Tonle Sap Lake, light trap, 17.V.2019, W. Rossi and V. Kong leg.; 1 ♂, Cambodia, Kampong Chhnang, Rolea B’ier District, Toulkrolanh Village, 12°13'31"N, 104°39'50"E, light trap, 7.XI.2018, W. Rossi and V. Kong leg.; 2 ♀♀, Cambodia, Banteay Meanchey Province, near Sisophon, Campus of the Mean Chey University, light trap, 22.X–23.XI.2019, P. Bun and W. Rossi leg.; 1 ♂, Cambodia, Khsam, Kampong Chhnang, 12°16'47"N, 104°39'28.6"E, light trap, 29.XI–3.XII.2019, W. Rossi and V. Kong leg. (CAl, CCa, CGi, BMNH, MCSNG).

#### Diagnosis.

A medium-sized (ABL: 15–18 mm) *Diplocheila* of the *polita* group in the subgenus Diplocheila (sensu Ball 1959). Among the species of this group having a sexsetose labrum, it is easily distinguished from *D. erwini* sp. nov. by the larger body size (15–18 mm vs 12–14 mm), from *D. laevigata* and *D. laevigotoides* by the more transverse pronotum (PW/PL = 1.38 vs 1.28–1.32), from *D. indus* by the hind angles of pronotum not protruding (externally protruding in *D. indus*) and from all these species by the morphology of the aedeagus.

#### Description.

***Habitus***: ABL: 15–18 mm (HT ♂ 15.6 mm). Body parallel-sided, moderately shiny, black with antennae and palpi piceous-brown (Fig. 4).

***Head***: almost quadrangular, robust, glabrous except for the supraorbital setae. Eyes markedly convex; a single supraorbital seta on each side. Dorsum with microsculpture not evident, only with scattered punctures visible at >100× magnification; frontal impressions short and superficial. Labrum symmetrically and deeply (LR = 0.72) emarginate, with six setigerous punctures on anterior margin (4 medial equidistant + 2 lateral on lobes). Clypeus trapezoid, distinctly concave anteriorly, with 1 seta at each anterolateral corner. Antennae moderately long, densely pubescent from segment 4, with terminal two articles surpassing base of pronotum; segments elongate, the second one short, as long as a half of first. Mandibles elongate, broad, approximately similar to one another (the left with apical cutting edge more concave), with scrobe well-defined and glabrous and apex blunt; terebral tooth triangular and prominent. Labial and maxillary palps fusiform, with apices narrowly truncate.

***Thorax***: pronotum smooth, with very faint isodiametric microsculpture evident at >200× magnification and with scattered punctures, transverse (PW/PL = 1.38), widest just above middle (Fig. 10). Disk moderately convex. Sides moderately rounded in anterior half, delicately sinuate backwards. Hind angles rounded obtuse, with a postero-lateral seta. Posterior margin rectilinear between basal impressions, which are linear and markedly impressed; anterior margin with front angles nearly obsolete. A single lateral seta on each side at anterior third. Lateral bead continuous, separated from the discal area by a narrow groove, only scarcely dilated before hind angles. Medial longitudinal impression fine, nearly reaching anterior and posterior margins; anterior transversal impression absent.

***Elytra***: moderately long (EL/EW = 1.65), parallel-sided, slightly convex and flattened on disk, widest at middle, with rounded shoulders and sides delicately sinuate before apex. Surface moderately shiny; microsculpture evident only at high magnification (>100×), consisting of fine, slightly transverse meshes. Epipleura without any distinct external plicae (“uncrossed epipleura”). Intervals moderately convex, smooth; striae deeply impressed on the whole length, delicately punctate. Parascutellar stria present; scutellar setigerous pore present at base of stria 1, just before conjunction with stria 2. Basal margin complete. Discal setigerous punctures absent; umbilicate series of setigerous punctures continuous, but punctures more widely spaced at middle. Hind wings fully developed.

***Ventral surface* (thorax and abdomen)**: prosternum and proepisterna glabrous and impunctate (only with very fine punctures). Metepisterna twice as long as their width at anterior side; metepimera narrow, nearly rectangular. Prosternal intercoxal process parallel-sided with blunt apex, delicately bordered. Abdominal ventrites IV–VI shiny but shagreened at sides, glabrous except one pair of subapical central setae; males with 2, females with 4 setae at apex of ventrite VII.

***Legs***: moderately slender. Posterior face of femora with 1 seta in profemora, 2 setae in mesofemora and metafemora. Metatrochanters glabrous and as long as one-third of metafemora. Protibial antennal cleaning organ well developed, with 2 clip setae. Protibiae robust, with 6 or 7 outer apical spines; mesotibiae with a group of setae at middle of inner face; metatibiae longitudinally furrowed at inner face. Dorsal face of tarsomeres smooth. Protarsomeres 1–3 of males moderately dilated, slightly asymmetrical; meso- and metatarsomeres not dilated in both sexes; tarsomere 5 ventrally glabrous, dorsally with 2 apical setae; claws smooth.

***Male genitalia***: median lobe of aedeagus short and moderately swollen before apex in lateral view (Fig. 22); apical lamella very short and apically rounded in dorsal view (Fig. 29), apex thick and very slightly bent downwards in lateral view. Ostium long, in dorsal position. Right paramere elongate and subtruncate at apex; left paramere conchoid.

#### Etymology.

This species is named after its collector, Walter Rossi, a world-renowned specialist in entomoparasitic fungi, as a token of our esteem and as a sign of gratitude for the gift to the authors of the specimens of the new species.

#### Distribution and ecology.

*Geographical distribution*: this species is recorded from Central and North-Western Cambodia (Fig. 33). *Life habits*: the specimens of the type series were collected by light trapping. No other data are available.

#### Remarks.

*Diplocheila walterrossii* sp. nov. belongs to the *D. polita* group (sensu Ball 1959), as it shares the characters distinguishing this group and, at first sight, is very similar in external morphology to *D. laevigata*. Nevertheless, at a deeper examination its aedeagus (Figs 22, 29) reveals evident morphological differences and is easy distinguished from that of *D. laevigata* (Figs 20, 26, 27), as well as from that of *D. erwini* sp. nov. (Figs 23, 30), both species sympatric and syntopic with *D. walterrossii* sp. nov. in Cambodia. Moreover, the new species shows a character almost unique in this group, as far as we know, that is the less enlarged male fore tarsi 1–3. For these reasons, *D. walterrossii* sp. nov. could represent a rather isolated species in the *D. polita* group, and its closest relatives remain uncertain.

### Diplocheila (Diplocheila) erwini sp. nov.

Taxon classificationAnimaliaColeopteraCarabidae

DC82C006-2D23-5FA0-8F17-B1D83A9DD9EB

http://zoobank.org/108A8C27-E70A-4322-AF94-73E2CE64DEAB

**Figure 5.**
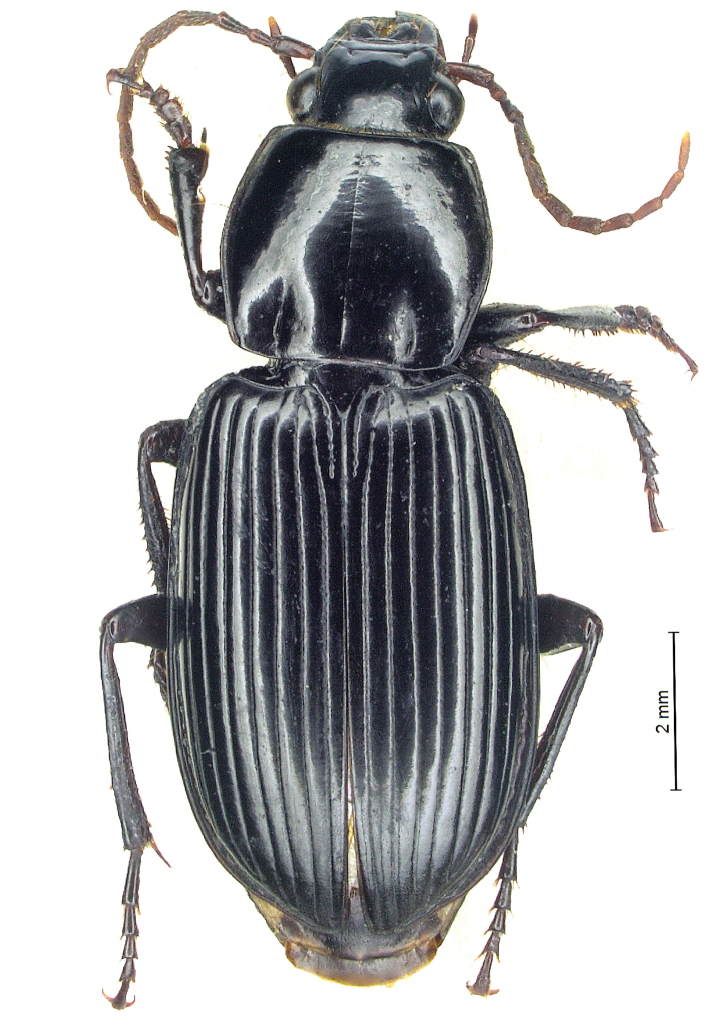
*Diplocheila erwini* sp. nov.: habitus of holotype.

Figures 5 , 11 , 17 , 23 , 30 , 33

#### Type locality.

Cambodia, Kampong Chhnang, Khom Domnatpopol, Tonle Sap Lake.

#### Material examined.

***Holotype***: ♂, Cambodia, Kampong Chhnang Province, Khom Domnatpopol, Tonle Sap Lake, 12°14'14"N, 104°41'15"E, (light trap), 21.V.2018, Rossi, Bernardi and Kong leg. (CAl).

***Paratypes***: (1 ♂ and 3 ♀♀); 1 ♀ same data as holotype; 1 ♂ Cambodia, Kampong Chhnang, Rolea B’ier District, Toulkrolanh Village, 12°13'31"N, 104°39'50"E, light trap, 7.XI.2018, W. Rossi and V. Kong leg.; 2 ♀♀, Cambodia, Kampong Chhnang, banks of Tonle Sap Lake, light trap, 17.V.2019, W. Rossi and V. Kong leg. (CAl, CGi).

#### Diagnosis.

A medium-sized to small *Diplocheila* (ABL: 12–14 mm) of the *polita* group in the subgenus Diplocheila (sensu Ball 1959). It is easily distinguished from the other species of the group with a sexsetose labrum (*D. indus*, *D. laevigata*, *D. laevigotoides*, and *D. walterrossii* sp. nov.) by the smaller body size (≤14 mm), the narrower and almost quadrangular pronotum (transverse in the four other species), with hind angles not protruding (protruding in *D. indus*), and by the morphology of the aedeagus.

#### Description.

***Habitus***: ABL: 12–14 mm (HT ♂ 13.5 mm). Body parallel-sided, moderately shiny, black with antennae and palpi piceous-brown (Fig. 5).

***Head***: almost quadrangular, glabrous except for the supraorbital setae, narrow in comparison with pronotum. Eyes markedly convex; a single supraorbital seta on each side. Dorsum with microsculpture not evident, only with scattered punctures visible at >100× magnification; frontal impressions short and superficial. Labrum symmetrically and deeply (LR = 0.78) emarginate, with six setigerous punctures on anterior margin (4 medial equidistant + 2 lateral on lobes). Clypeus trapezoid, distinctly concave anteriorly, with 1 seta on each side at anterolateral corner. Antennae moderately long, densely pubescent from segment 4, with terminal 2 articles surpassing base of pronotum; segments elongate, the second one short, as long as a half of first. Mandibles elongate, broad, approximately similar each another (the left with apical cutting edge more concave), with scrobe well-defined and glabrous and apex blunt; terebral tooth triangular and prominent. Labial and maxillary palps fusiform, with apices narrowly truncate.

***Thorax***: pronotum smooth, with very faint microsculpture evident at >200× magnification and with scattered punctures, subquadrate (PW/PL = 1.18), widest at middle (Fig. 11). Disk moderately convex. Sides from rectilinear to hardly rounded in anterior half; rectilinear or very slightly sinuate backwards. Hind angles rounded obtuse, provided with a postero-lateral seta. Posterior margin rectilinear between basal impressions, which are linear and markedly impressed; anterior margin with front angles nearly obsolete. A single lateral seta on each side just above middle. Lateral bead continuous, separated from the discal area by a narrow groove, only scarcely dilated before hind angles. Medial longitudinal impression fine, nearly reaching anterior and posterior margins; anterior transversal impression absent.

***Elytra***: moderately long (EL/EW = 1.59), parallel-sided, slightly convex and flattened on disk, widest at middle, with rounded shoulders and sides delicately sinuate before apex. Surface moderately shiny; microsculpture evident only at high magnification (>100×), consisting of fine, slightly transverse meshes. Epipleura without any distinct external plicae (“uncrossed epipleura”). Intervals moderately convex, smooth; striae deeply impressed on the whole length, distinctly punctate. Parascutellar stria present; scutellar setigerous pore present at base of stria 1, just before conjunction with stria 2. Basal margin complete. Discal setigerous punctures absent; umbilicate series of setigerous punctures continuous, not interrupted at middle. Hind wings fully developed.

***Ventral surface* (thorax and abdomen)**: prosternum and proepisterna glabrous and impunctate (only with very fine punctures). Metepisterna as long as twice the width of anterior side; metepimera large, broadly rounded. Prosternal intercoxal process widely rounded and bordered at apex. Abdominal ventrites IV–VI shiny but shagreened at sides, glabrous except one pair subapical central setae; males with 2, females with 4 marginal setae at apex of ventrite VII.

***Legs***: moderately slender. Posterior face of femora with 1 seta in profemora, 2 in mesofemora and metafemora. Metatrochanters glabrous and slightly shorter than half length of metafemora. Protibial antennal cleaning organ well developed, with 2 clip setae. Protibiae robust, with 4 or 5 outer apical spines; mesotibiae with a group of setae at middle of inner face; metatibiae longitudinally furrowed at inner face. Dorsal face of tarsomeres smooth. Male protarsomeres 1–3 distinctly dilated, slightly asymmetrical; meso- and metatarsomeres not dilated in both sexes; tarsomere 5 ventrally glabrous, dorsally with 2 apical setae; claws smooth.

***Male genitalia***: median lobe of aedeagus short and markedly swollen before apex in lateral view (Fig. 23); the apical lamella shortly triangular in dorsal view, with blunt tip (Fig. 30), apex very slightly bent downwards in lateral view. Ostium long, in dorsal position. Right paramere oval; left paramere conchoid.

#### Etymology.

This species is named, as a token of our esteem, after our late colleague Terry Erwin, a world-renowned specialist in world and tropical Carabidae.

#### Distribution and ecology.

*Geographical distribution*: this species is recorded only from the extreme south banks of the Tonle Sap Lake, Kampong Chhnang Province, Cambodia. It seems to have a more restricted distribution than *D. walterrossii* sp. nov., which has been recorded from the same site as well as from other two localities in north-western Cambodia (Fig. 33). *Life habits*: the specimens of the type series were collected on lake banks by light trapping. No other data are available.

#### Remarks.

It is difficult to assess the closest relatives of *D. erwini* sp. nov. It belongs to the *D. polita* group (sensu Ball 1959), as it shares the characters distinguishing this group and, moreover, its aedeagus is very similar to that of *D. polita* (see pl. V, fig. 72a in Ball 1959). *Diplocheila erwini* sp. nov., on the other hand, is easily distinguished from *D. polita* which has a quadrisetose labrum and pronotum more transverse (PW/PL = 1.31 according to Fig. 26 in Plate II in Ball 1959), as well as a larger body size (ABL = 13.4–18.4) (Ball 1959).

**Figures 6–11.**
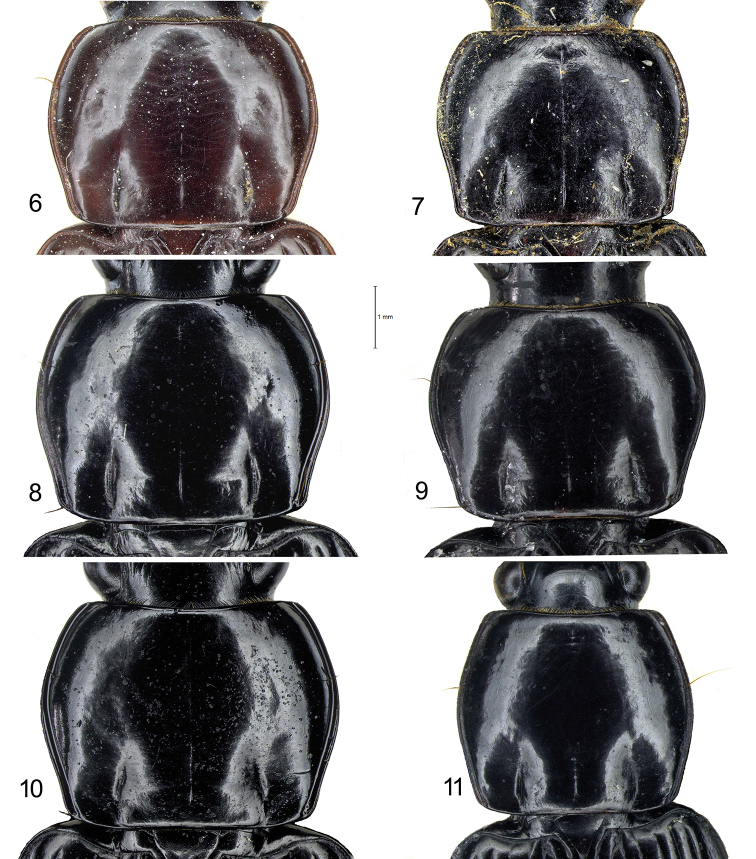
Pronotum of *Diplocheila* species **6***D. laevigata*HT**7***D. laevigotoides*HT**8***D. laevigata* from Cambodia **9***Diplocheila* sp. from Thailand **10***D. walterrossii* sp. nov. HT**11***D. erwini* sp. nov. HT.

**Figures 12–17.**
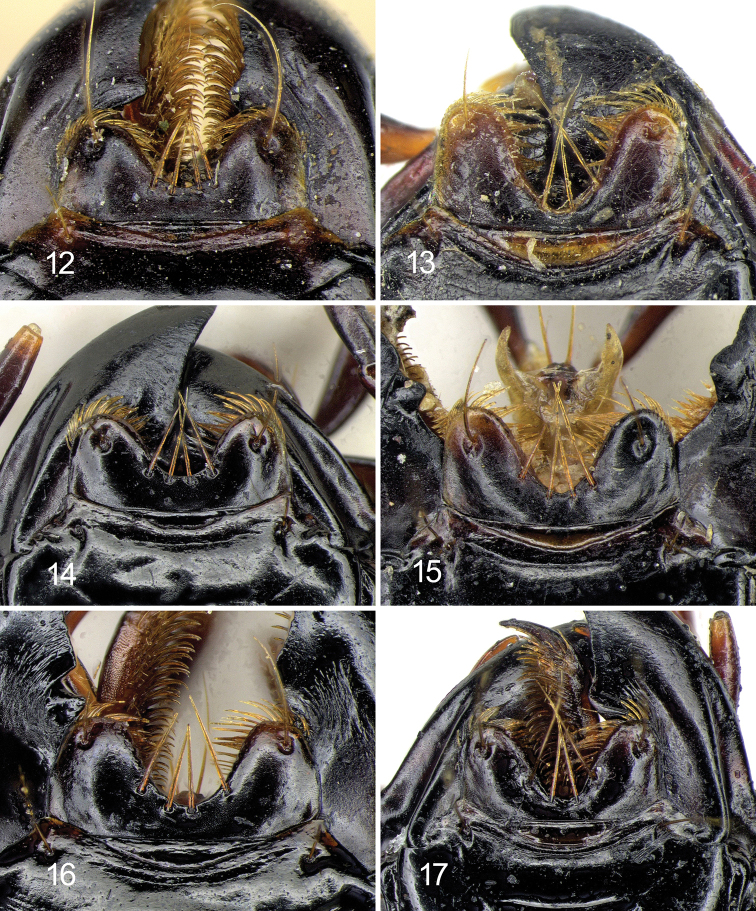
Labrum of *Diplocheila* species **12***D. laevigata*HT**13***D. laevigotoides*HT**14***D. laevigata* from Cambodia **15***Diplocheila* sp. from Thailand **16***D. walterrossii* sp. nov. HT**17***D. erwini* sp. nov. HT.

**Figures 18–19.**
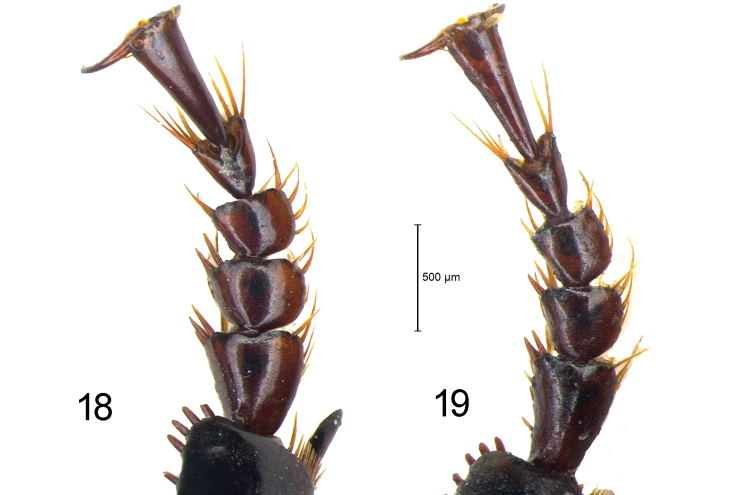
Male protarsi of *Diplocheila* species **18***D. laevigata* from Cambodia **19***D. walterrossii* sp. nov. HT.

### Key to the species of *Diplocheila* Brullé, 1834 recorded from Cambodia (including *D. laevigotoides*)^1^

**Table:** 

1	Head with 1 supraorbital setiferous pore on each side	**2**
–	Head with 2 supraorbital setiferous pores on each side	**6**
2	Labrum with 4 setae (2 medial + 2 lateral). Anterior margin of clypeus almost straight. Antennal scape clavate, longer than 3 times its width	**D. (Diplocheila) distinguenda (LaFerté-Sénectère, 1851)**
–	Labrum with 6 setae (4 medial + 2 lateral). Anterior margin of clypeus more or less emarginate. Antennal scape normal, shorter than 3 times its width	**3**
3	Pronotum subquadrate (W/L = 1.18). Smaller body size (12–14 mm)	**D. (Diplocheila) erwini sp. nov.**
–	Pronotum distinctly transverse (W/L = 1.27–1.38). Larger body size (>14 mm)	**4**
4	Elytral striae nearly impunctate. Labrum deeply emarginate (RL = 0.80) (Fig. 13). Pronotum sides markedly sinuate backwards (Fig. 7). Anterior margin of clypeus distinctly concave (Fig. 13). Probably not occurring in Cambodia	[**D. (Diplocheila) laevigotoides Jedlička, 1936**]
–	Elytral striae punctate. Labrum less deeply emarginate (RL = 0.63–0.72) (Figs 12, 14, 15). Pronotum sides less abruptly sinuate backwards (Figs 6, 8, 9, 10). Anterior margin of clypeus more or less concave	**5**
5	Pronotum more transverse (W/L = 1.38) (Fig. 10). Male fore tarsi moderately dilated (Fig. 19). Labrum more deeply emarginate (RL = 0.72) (Fig. 16). Anterior margin of clypeus distinctly concave (Fig. 16). Apical lamella of the median lobe of aedeagus very short (Figs 22, 29)	**D. (Diplocheila) walterrossii sp. nov.**
–	Pronotum less transverse (W/L = 1.28–1.32) (Figs 6, 8). Male fore tarsi markedly dilated (Fig. 18). Labrum moderately emarginate (RL = 0.63) (Figs 12, 14). Anterior margin of clypeus hardly concave (Figs 12, 14). Apical lamella of the median lobe of aedeagus shortly triangular, with blunt apex (Figs 20, 26, 27)	**D. (Diplocheila) laevigata (Bates, 1892)**
6	Labrum with 6 setae (4 medial + 2 lateral), symmetrical, deeply emarginate with lateral lobes narrow and acute at apex. Pronotum and elytra matt. Smaller body size (<18 mm)	**D. (Neorembus) latifrons ssp. latifrons (Dejean, 1831)**
–	Labrum with 4 setae (2 medial + 2 lateral), only moderately emarginate. Pronotum and elytra more or less glossy. Larger body size (>20 mm)	**7**
7	Elytral striae well impressed. Labrum symmetrical, with lateral lobes rounded at apex	**D. (Diplocheila) colossus (Bates, 1892)**
–	Elytral striae shallowly impressed, nearly obsolete. Labrum asymmetrical, with lateral lobes pointed at apex and left lobe larger than right	**D. (Submera) laevis (Lesne, 1896)**

**Figures 20–25.**
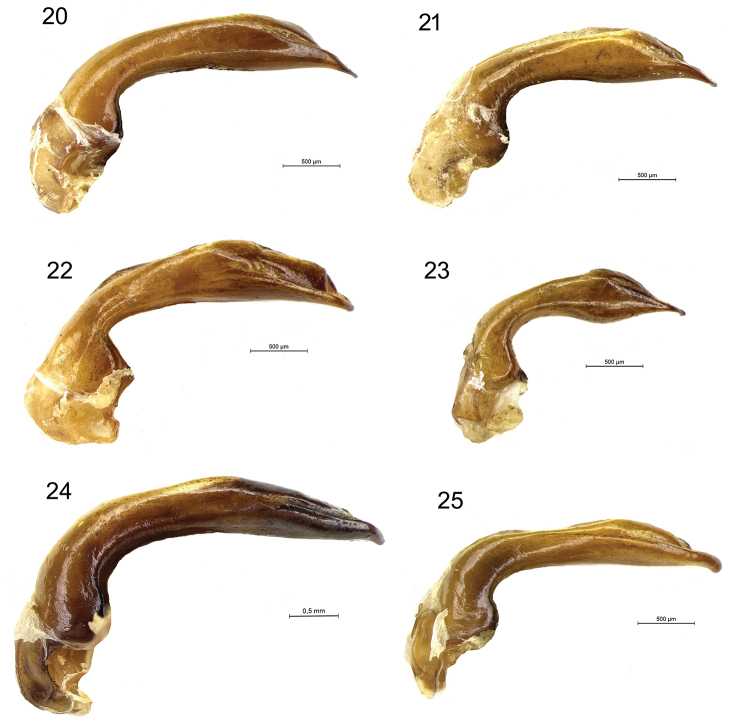
Aedeagus in lateral view of *Diplocheila* species **20***D. laevigata* from Cambodia **21***Diplocheila* sp. from Thailand **22***D. walterrossii* sp. nov. HT**23***D. erwini* sp. nov. HT**24***D. laevis* from Cambodia **25***D. latifrons* from Cambodia.

**Figures 26–32.**
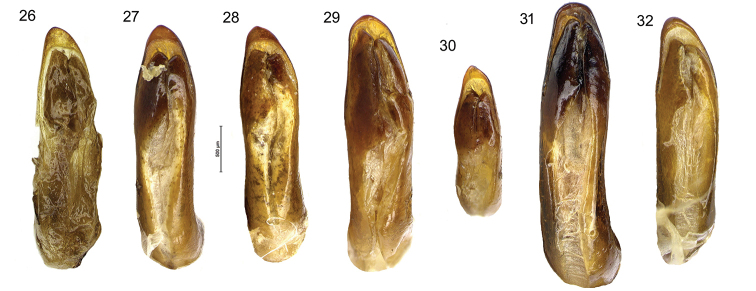
Aedeagus in dorsal view of *Diplocheila* species **26***D. laevigata*HT**27***D. laevigata* from Cambodia **28***Diplocheila* sp. from Thailand **29***D. walterrossii* sp. nov. HT**30***D. erwini* sp. nov. HT**31***D. laevis* from Cambodia **32***D. latifrons* from Cambodia.

**Figure 33.**
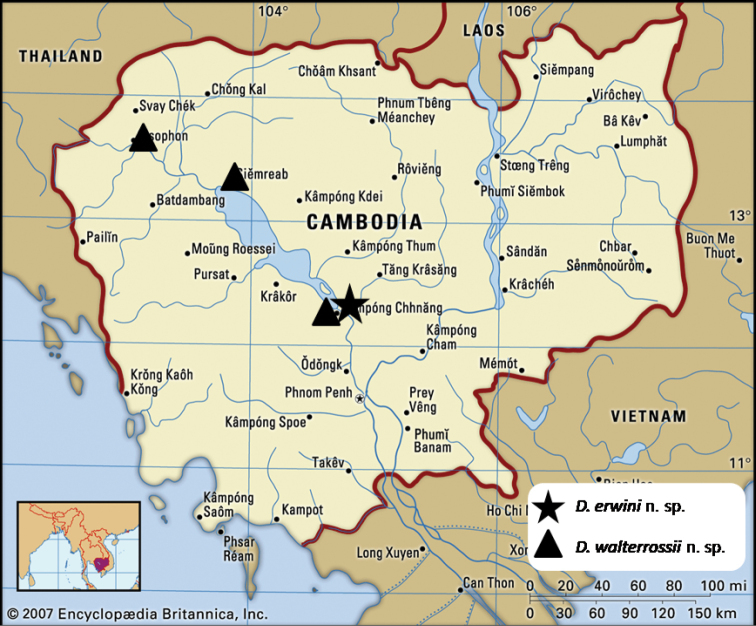
Distribution map of *Diplocheila walterrossii* sp. nov. and *D. erwini* sp. nov. in Cambodia.

## Conclusions

Seven species of the genus *Diplocheila* are currently known from Cambodia. Two new species (*D. erwini* sp. nov. and *D. walterrossii* sp. nov.) are here added to its fauna, and *D. laevis* is recorded for the first time from this country. As the adults are macropterus and probably good fliers, no species are likely steno-endemic, although *D. erwini* sp. nov. and *D. walterrossii* sp. nov. are only known from Cambodia to date.

The discovery of a new species (*D. walterrossii* sp. nov.) with external morphology very similar to *D. laevigata* drove us to study and compare *D. laevigata* and *D. laevigotoides*, which were often misunderstood and misidentified in the past. The examination of the holotypes of these species confirmed their validity and the status of *D. walterrossii* sp. nov., providing new morphological information useful for the correct identification of the three taxa. Finally, the study of various specimens from countries of the Oriental Region other than Cambodia convinced us of the possibility of additional new species, similar to *D. laevigata* in external morphology and therefore confused with it in the past, awaiting discovery and description.

## Supplementary Material

XML Treatment for Diplocheila (Neorembus) latifrons ssp. latifrons

XML Treatment for Diplocheila (Submera) laevis

XML Treatment for Diplocheila (Diplocheila) laevigata

XML Treatment for Diplocheila (Diplocheila) laevigotoides

XML Treatment for Diplocheila (Diplocheila) walterrossii

XML Treatment for Diplocheila (Diplocheila) erwini

## Footnotes

1 Modified from Ball 1959 and Lafer and Kataev 2008.
